# Asthma control using fluticasone propionate/salmeterol in Asian and non-Asian populations: a post hoc analysis of the GOAL study

**DOI:** 10.1186/s12890-017-0410-x

**Published:** 2017-04-28

**Authors:** Jean Bousquet, Neil Barnes, Michael Gibbs, Nadeem Gul, Susan A Tomkins, Xin Zhou, Young-Joo Cho, Hae-Sim Park, William Busse, Nanshan Zhong

**Affiliations:** 1 Fondation MACVIA-LR, Contre les Maladies Chroniques pour un Vieillissement Actif en Languedoc-Roussillon, European Innovation Partnership on Active and Healthy Ageing Reference Site, University of Montpellier, Montpellier, France; 2 INSERM, VIMA: Ageing and Chronic Diseases, Epidemiological and Public Health Approaches, U1168, Paris, France; 3 UVSQ, UMR-S 1168, Université Versailles St-Quentin-en-Yvelines, Paris, France; 4 Global Respiratory Franchise, GSK House, Brentford, Middlesex UK; 5 William Harvey Institute, Barts and the London School of Medicine and Dentistry, London, UK; 6 Quantitative Science, Clinical Statistics, GSK, Stockley Park, Middlesex, UK; 7 Shanghai First People’s Hospital, Shanghai, China; 8 Ewha Womans University, School of Medicine, Seoul, South Korea; 9 Ajou University Medical Center, Suwon, South Korea; 10 University of Wisconsin, School of Medicine and Public Health, Madison, WI USA; 11 State Key Laboratory of Respiratory Disease, Guangzhou Institute of Respiratory Disease, First Affiliated Hospital of Guangzhou Medical University, Guangzhou, China

**Keywords:** Asian patients, Asthma, Asthma control, Asthma treatment, Fluticasone propionate/salmeterol, ICS/LABA treatment

## Abstract

**Background:**

To analyse the efficacy of fluticasone propionate (FP) alone and combined with salmeterol (SAL) in achieving guideline-defined asthma control in Asian patients.

**Methods:**

A post hoc analysis of the GOAL study in which patients were stratified by prior-medication use into inhaled corticosteroid (ICS)-naïve (Stratum [S] 1), low-dose ICS (S2), and medium-dose ICS (S3), and randomised to receive FP/SAL or FP. Doses were stepped-up every 12 weeks until Totally Controlled asthma or maximum dose was reached (PhI) and then maintained until study end (PhII). The primary endpoint was the proportion of patients achieving Well-Controlled asthma during PhI. Additional endpoints included Total Control and adverse events. Asian and non-Asian patients were analysed separately.

**Results:**

In Asian patients in PhI, 74% (*n* = 87/118) in S1 achieved Well-Controlled asthma with FP/SAL versus 74% (*n* = 89/121) with FP alone (*p* = 0.839); corresponding values were 76% (*n* = 81/107) versus 60% (*n* = 62/104; *p* = 0.005) in S2, and 58% (*n* = 59/102) versus 43% (*n* = 41/95; *p* = 0.015) in S3. More patients in all three strata achieved Totally Controlled asthma with FP/SAL versus FP alone. Control was achieved more rapidly and with lower ICS doses with FP/SAL versus FP. A high proportion of patients who achieved control during PhI maintained control during PhII. Similar trends were found in non-Asian patients. No new safety concerns were identified.

**Conclusions:**

A greater proportion of Asian patients (S2 and S3, for Well-Controlled; all strata, for Totally Controlled) achieved guideline-defined asthma control with FP/SAL versus FP alone. High proportions of Asian patients in S1 achieved Well-Controlled asthma in both treatment groups.

**Electronic supplementary material:**

The online version of this article (doi:10.1186/s12890-017-0410-x) contains supplementary material, which is available to authorized users.

## Background

In the past two decades, asthma guidelines have been revised to emphasise management based on the control of symptoms and exacerbations rather than classification of the patient by disease severity [1, 2]. The current Global Initiative for Asthma (GINA) guidelines describe the level of asthma symptom control as well-controlled, partly controlled and uncontrolled [1].

The Gaining Optimal Asthma controL (GOAL) study was the first large study to examine whether guideline-defined asthma control can be achieved in patients who were uncontrolled on either short-acting β2-agonist (SABA) alone or low- to medium-dose inhaled corticosteroid (ICS) [3]. GOAL was a 1-year, randomised, multinational trial which included 3,421 patients from 44 countries [3]. The study evaluated fluticasone propionate (FP) in combination with salmeterol (SAL) (FP/SAL) compared with FP alone, and demonstrated that guideline-defined asthma control could be achieved in the majority of patients [3].

The large size and multinational nature of GOAL allows for analysis of population subgroups, for example, by ethnicity. The relatively few clinical trials evaluating asthma control in Asian patients have demonstrated that effective control can be achieved with usual therapies of ICS alone or a combination of ICS/long-acting β_2_-agonist (LABA) [4–6]. However, two large, population-based surveys in Asia have shown that good asthma control is not always achieved in this geographic area, despite the availability of effective treatments [7–9]. In the Asthma Insights and Reality in Asia-Pacific (AIRIAP) survey, a high proportion of patients had poorly-controlled asthma according to GINA criteria [7]. Approximately half of patients surveyed had experienced daytime or night-time asthma symptoms over the past month, and a similar proportion had required emergency care over the past year [7]. The REcognise Asthma and LInk to Symptoms and Experience (REALISE) Asia study reported uncontrolled asthma in half the patients surveyed and only partial control in a further one-third [9]. Two out of three patients missed work because of asthma, and even patients reported as having controlled asthma had experienced acute exacerbations during the previous year [8].

The underlying reasons for poor control in patients with asthma are unknown. Several studies have demonstrated that lung function measures in Asian populations are lower than those in Caucasian populations [10–13]. How this difference may impact on the level of asthma control that can be achieved with treatment in Asian patients is unknown. To help answer this question, we conducted a post hoc subgroup analysis of Asian patients in the GOAL study, using the composite asthma control criteria used in the original global population. The primary objective of this post hoc analysis was to evaluate the efficacy of FP/SAL compared with FP alone in achieving guideline-defined asthma control in Asian patients. Additionally, data from the non-Asian population are presented to provide a descriptive comparison with the results from the Asian population.

## Methods

### Study design

This was a post hoc subgroup analysis of the randomised, double-blind, parallel-group GOAL study (GSK identifier: SAM40027). The design and methodology of the GOAL study have been published in detail [3] (Additional file 1). Patients were recruited between December 2000 and December 2001 and stratified according to their medication use in the 6 months prior to screening. Stratum 1 included patients who were ICS-naïve, stratum 2 included patients who received low-dose ICS treatment (≤500 μg daily of beclomethasone dipropionate or equivalent), and stratum 3 included patients who received medium-dose ICS treatment (>500– ≤1000 μg daily of beclomethasone dipropionate or equivalent).

Patients were randomised (1:1) to receive either FP/SAL or FP, stratified by baseline therapy. The starting doses (taken twice daily) were FP/SAL 100/50 μg or FP 100 μg (patients in stratum 1 and stratum 2) or FP/SAL 250/50 μg or FP 250 μg (patients in stratum 3). The study was divided into two phases. In Phase I, doses were “stepped-up” every 12 weeks until either Total Control (as defined by the GINA and National Institutes of Health [NIH] Guidelines at the time of the original study [14]; Additional file 2: Table S1) was achieved or the maximum dose (FP/SAL 500/50 μg or FP 500 μg) was reached. Patients in stratum 1 and stratum 2 had up to three treatment steps (step 1: FP/SAL 100/50 μg or FP 100 μg; step 2: FP/SAL 250/50 μg or FP 250 μg; step 3: FP/SAL 500/50 μg or FP 500 μg) while patients in stratum 3 had up to two steps (step 1: FP/SAL 250/50 μg or FP 250 μg; step 2: FP/SAL 500/50 μg or FP 500 μg). In Phase II (reached either after achieving Total Control in Phase I or after 12 weeks on the maximum dose), patients remained on the dose that achieved Total Control or the maximum dose until the end of the 1-year double-blind study period. Patients who failed to achieve Total Control by the end of Phase II entered a 4-week, open-label phase during which they received oral prednisolone (0.5 mg/kg up to 60 mg per day for 10 days) and FP/SAL 500/50 μg twice daily.

### Study population

Only patients who did not demonstrate Well-Controlled asthma for at least 2 weeks of the 4-week run-in period were eligible for inclusion in GOAL (Additional file 2: Table S1). For this post hoc subgroup analysis, the Asian population was defined as patients of Chinese, Japanese, Korean, Thai, Malay or Filipino origin living in South East Asia (China, Japan, Hong Kong, Malaysia, Philippines, Singapore, South Korea, Taiwan, Thailand) during the study. The non-Asian population included all patients in the intent-to-treat (ITT) population who were not in the Asian subgroup (full inclusion criteria of the GOAL study can be found in the Additional file 1).

### Study endpoints

The primary endpoint of the GOAL study and of this post hoc subgroup analysis was the proportion of patients achieving Well-Controlled asthma with FP/SAL compared with FP alone during Phase I. The proportion of patients achieving Total Control of asthma was also evaluated. Asthma control was defined according to composite measures based on the GINA/NIH criteria developed for the GOAL study [3, 14], as shown in Additional file 2: Table S1. Well-Controlled asthma and Total Control of asthma were achieved if the patient recorded 7 out of 8 consecutive assessment weeks with Well-Controlled asthma or Total Control of asthma, respectively. Additional endpoints included: the ICS dose at which Well-Controlled asthma and Total Control of asthma was achieved; the cumulative probabilities from analysis of time to achieve the first week of Well-Controlled asthma and Total Control of asthma; the maintenance of Well-Controlled asthma and Total Control of asthma status from Phase I until the end of Phase II; the stability of weekly control during Phase II; rate of exacerbations (requiring oral corticosteroids, hospitalisations or emergency visits); and morning predose clinic forced expiratory volume in 1 s (FEV_1_) and asthma quality of life (QoL) as assessed using the Asthma Quality of Life Questionnaire (AQLQ) [15]. The incidence of adverse events (AEs) was also monitored.

### Calculation of the sample size

Sample size calculations were performed for the original GOAL study [3]; however, as the current analysis was post hoc, no further sample size calculations were required and therefore all analyses are descriptive only, including the calculated p-values.

### Statistical analysis

The ITT population, defined as all patients who were randomised and received at least one dose of a study drug, was used for all analyses. Using data from a Caucasian-only subpopulation (comprising approximately 90% of the non-Asian patients in the GOAL study), the primary endpoint was analysed and found to have little difference compared with the overall non-Asian population, so data from the non-Asian population are presented here.

The statistical analysis of the proportion of patients who achieved Well-Controlled asthma and Total Control of asthma was performed using logistic regression with covariates for baseline FEV_1_, sex, age and treatment group. The analysis of the ICS dose at which Well-Controlled asthma and Total Control of asthma was first achieved was performed using proportional odds logistic regression on dose with the same covariates. The effects of treatment on exacerbation rates were determined using Poisson regression with generalised estimating equations with the same covariates (treatment differences were expressed as the ratio of FP/SAL over FP, with treatment ratios greater than 1 in favour of FP/SAL). Time to achieve Well-Controlled asthma and Total Control of asthma was assessed using inverse Kaplan–Meier plots with Log-Rank tests. The statistical analysis of mean change in clinic FEV_1_ (L) was performed using analysis of covariance with covariates for baseline FEV_1_, sex, age and treatment group (treatment differences were expressed as a difference of FP/SAL from placebo in FEV_1_ [L], with positive treatment differences in favour of FP/SAL).

Analyses were completed using SAS 9.3 (Cary, NC, USA).

## Results

### Study population

**Fig. 1:**
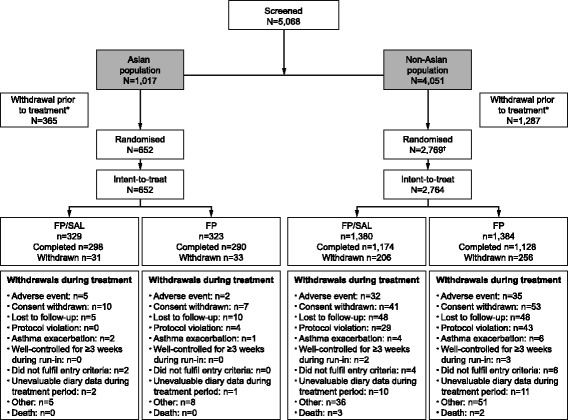
Patient disposition. *Includes those who were classified as screen or run-in failures; ^†^includes five patients who were excluded from the intent-to-treat population as they did not take a dose of the study drug. *FP* fluticasone propionate, *SAL* salmeterol

A total of 5,068 patients were screened and 3,421 qualified for inclusion in the primary study [3]. Of these patients, 652 Asian patients and 2,764 non-Asian patients were randomised and treated and therefore included in this post hoc subgroup analysis (Fig. 1).

The demographics and baseline characteristics of the two populations were generally similar across the different strata and treatment groups (Table 1). However, compared with the Asian patients, non-Asian patients had a numerically greater mean height, weight and percent predicted pre-FEV_1_ (Table 1). Patient race is shown in Additional file 3: Table S2.

**Table 1:** Patient demographics and baseline characteristics (ITT population)

	Asian population						Non-Asian population					
	S1		S2		S3		S1		S2		S3	
	FP/SAL	FP	FP/SAL	FP	FP/SAL	FP	FP/SAL	FP	FP/SAL	FP	FP/SAL	FP
	n = 120	n = 122	n = 107	n = 104	n = 102	n = 97	n = 428	n = 428	n = 478	n = 474	n = 474	n = 482
Age, mean (SD), years^a^	37.2 (14.4)	39.1 (14.4)	41.9 (15.4)	41.4 (13.5)	45.8 (14.3)	43.1 (12.4)	35.8 (16.0)	35.6 (15.8)	40.0 (16.6)	40.1 (17.2)	43.7 (16.2)	42.6 (16.3)
Female, n (%)^a^	67 (56)	75 (61)	62 (58)	61 (59)	53 (52)	63 (65)	245 (57)	241 (56)	278 (58)	288 (61)	276 (58)	279 (58)
Height, mean (SD), cm^a^	161.9 (9.5)	158.6 (8.8)	161.3 (8.9)	162.7 (8.8)	161.6 (9.5)	161.3 (7.9)	167.8 (9.8)	167.7 (10.1)	166.5 (10.2)	166.5 (10.2)	167.1 (10.1)	166.6 (10.3)
Weight, mean (SD), kg^a^	60.8 (12.9)	58.6 (11.7)	61.5 (11.3)	61.8 (11.1)	63.2 (14.0)	61.7 (10.6)	72.4 (17.6)	71.8 (17.9)	72.6 (16.7)	72.7 (17.9)	74.5 (16.5)	73.4 (16.4)
BMI, mean (SD)^a^	23.15 (4.57)	23.25 (3.94)	23.59 (3.69)	23.28 (3.41)	24.07 (4.33)	23.68 (3.37)	25.63 (5.72)	25.41 (5.73)	26.16 (5.52)	26.10 (5.56)	26.59 (5.09)	26.53 (5.46)
Mean FEV_1_ at visit 2 (SD), L^b^	2.08 (0.69) *n = 118*	1.92 (0.68) *n = 121*	1.91 (0.64) *n = 107*	1.97 (0.58) *n = 104*	1.93 (0.75) *n = 102*	2.04 (0.68) *n = 95*	2.61 (0.88) *n = 421*	2.69 (0.82) *n = 423*	2.53 (0.82) *n = 476*	2.46 (0.82) *n = 473*	2.36 (0.81) *n = 466*	2.38 (0.79) *n = 472*
Mean % predicted^c^ FEV_1_ at visit 2 (SD), %^b^	68.6 (16.6) *n = 118*	67.6 (17.0) *n = 121*	66.7 (16.8) *n = 107*	67.0 (15.6) *n = 104*	67.3 (17.1) *n = 102*	71.1 (16.8) *n = 95*	78.8 (18.6) *n = 421*	81.8 (18.0) *n = 423*	80.4 (17.6) *n = 476*	79.3 (18.2) *n = 473*	76.2 (18.6) *n = 466*	77.1 (17.6) *n = 472*
Median pre-randomisation reversibility, (min., max.), %	21.4 (10.5, 95.2) *n = 91*	20.0 (9.0, 112.5) *n = 98*	25.0 (2.7, 124.7) *n = 87*	24.0 (11.1, 78.9) *n = 80*	25.3 (4.2, 92.0) *n = 63*	20.2 (8.3, 68.6) *n = 67*	23.8 (1.0, 95.7) *n = 318*	22.0 (2.5, 97.0) *n = 324*	21.4 (−0.6, 123.6) *n = 346*	21.6 (3.7, 111.5) *n = 346*	22.2 (−0.4, 115.3) *n = 356*	21.9 (3.1, 224.0) *n = 366*
Mean am PEF (SD), L/min	310.5 (76.3) *n = 120*	299.0 (72.7) *n = 122*	312.3 (92.3) *n = 107*	311.0 (70.9) *n = 104*	320.1 (84.2) *n = 102*	327.9 (90.5) *n = 97*	353.8 (92.9) *n = 427*	358.4 (93.8) *n = 427*	356.9 (98.0) *n = 478*	350.7 (96.5) *n = 474*	350.4 (100.8) *n = 473*	352.5 (97.0) *n = 482*
Mean % predicted^b^ am PEF (SD), %	71.1 (14.1) *n = 120*	71.9 (14.4) *n = 122*	74.1 (18.4) *n = 107*	72.7 (15.9) *n = 104*	74.8 (14.4) *n = 102*	77.5 (17.2) *n = 97*	76.9 (14.5) *n = 427*	77.9 (14.1) *n = 427*	79.4 (15.3) *n = 478*	79.3 (16.2) *n = 474*	78.4 (16.2) *n = 473*	79.2 (16.0) *n = 482*
Mean daily rescue usage, (SD)	1.9 (1.6) *n = 120*	1.7 (1.3) *n = 122*	1.7 (1.3) *n = 107*	1.5 (1.1) *n = 103*	1.5 (1.5) *n = 102*	1.6 (1.4) *n = 97*	2.0 (1.7) *n = 426*	1.8 (1.5) *n = 428*	1.8 (1.5) *n = 478*	1.8 (1.5) *n = 473*	2.0 (1.4) *n = 473*	2.0 (1.4) *n = 482*
Median night-time awakenings, (min., max.)	0.5 (0, 3) *n = 120*	0.5 (0, 3) *n = 122*	0.2 (0, 3) *n = 107*	0.2 (0, 2) *n = 104*	0.2 (0, 2) *n = 102*	0.3 (0, 4) *n = 95*	0.4 (0, 4) *n = 425*	0.3 (0, 4) *n = 427*	0.2 (0, 3) *n = 476*	0.2 (0, 3) *n = 471*	0.3 (0, 4) *n = 471*	0.2 (0, 3) *n = 479*
Mean asthma symptom score, (SD)	1.9 (0.8) *n = 120*	1.7 (0.8) *n = 121*	1.7 (0.9) *n = 107*	1.6 (0.8) *n = 104*	1.6 (0.9) *n = 102*	1.8 (1.0) *n = 97*	1.8 (0.8) *n = 427*	1.7 (0.9) *n = 428*	1.8 (0.9) *n = 478*	1.8 (0.9) *n = 473*	2.0 (0.9) *n = 472*	1.9 (0.9) *n = 482*
Patients with atopy, n (%)^a^	54 (45)	46 (38)	54 (50)	59 (57)	62 (61)	46 (47)	259 (61)	253 (59)	298 (62)	278 (59)	300 (63)	290 (60)
Patients with unknown atopy, n (%)^a^	8 (7)	10 (8)	13 (12)	9 (9)	18 (18)	10 (10)	46 (11)	34 (8)	31 (6)	25 (5)	32 (7)	30 (6)

*am* morning, *BMI* body mass index, *FEV*_*1*_ forced expiratory volume in 1 s, *FP* fluticasone propionate, *FP/SAL* fluticasone propionate/salmeterol, *ICS* inhaled corticosteroids, *ITT* intent-to-treat, *max*. maximum, *min.* minimum, *PEF* peak expiratory flow, *S1* patients who were ICS naïve at study entry, *S2* patients who received low-dose ICS treatment prior to study entry, *S3* patients who received medium-dose ICS treatment prior to study entry, *SD* standard deviation

^a^Number of patients with data to analyse for this variable as in the column heading, apart from weight and BMI in the non-Asian/S2/FP cells, where *n* = 473 (italicised *n* = in other rows are the number of patients with data available for analysis in this variable); ^b^pre-bronchodilator; ^c^predicted values calculated from European Community for Coal and Steel (ECCS) values

### Achievement of guideline-defined asthma control

#### Overall control

In Phase I, the same percentage of Asian patients in stratum 1 achieved Well-Controlled asthma with FP/SAL (74% [87/118]) and with FP alone (74% [89/121]) (odds ratio [OR]: 0.94; 95% confidence interval [95% CI]: 0.52, 1.70; *p* = 0.839). More Asian patients in stratum 2 and stratum 3 achieved Well-Controlled asthma with FP/SAL than with FP alone (stratum 2: 76% [81/107] vs 60% [62/104], respectively [OR: 2.44; 95% CI: 1.31, 4.56; *p* = 0.005]; stratum 3: 58% [59/102] vs 43% [41/95], respectively [OR: 2.08; 95% CI: 1.15, 3.76; *p* = 0.015]). For the non-Asian population, more patients in all three strata achieved Well-Controlled asthma with FP/SAL than with FP alone (stratum 1: 70% [296/421] vs 63% [267/423], respectively [OR: 1.41; 95% CI: 1.06, 1.88; *p* = 0.020]; stratum 2: 67% [319/476] vs 51% [240/473] [OR: 2.00; 95% CI: 1.53, 2.61; *p* < 0.001]; stratum 3: 49% [229/466] vs 31% [147/472] [OR: 2.19; 95% CI: 1.68, 2.87; *p* < 0.001]).

In Phase I, more patients in all three strata achieved Total Control of asthma with FP/SAL compared with FP alone in both the Asian and non-Asian populations. In Asian patients in stratum 1, Total Control was achieved in 42% (49/118) of patients with FP/SAL and 28% (34/121) with FP (OR: 1.77; 95% CI: 1.02, 3.08; *p* = 0.043), in stratum 2 this was achieved in 36% (39/107) versus 25% (26/104), respectively (OR: 1.86; 95% CI: 1.00, 3.43; *p* = 0.049), and in stratum 3 this was achieved in 26% (27/102) versus 6% (6/95), respectively (OR: 7.16; 95% CI: 2.65, 19.35; *p* < 0.001). In non-Asian patients in stratum 1, Total Control was achieved in 42% (176/421) of patients with FP/SAL and 32% (135/423) with FP (OR: 1.56; 95% CI: 1.18, 2.07; *p* = 0.002), in stratum 2 this was achieved in 32% (150/476) versus 19% (88/473), respectively (OR: 1.99; 95% CI: 1.47, 2.70; *p* < 0.001), and in stratum 3 this was achieved in 17% (79/466) versus 8% (37/472), respectively (OR: 2.42; 95% CI: 1.60, 3.67; *p* < 0.001).

#### ICS dose at which control was achieved

**Fig. 2:**
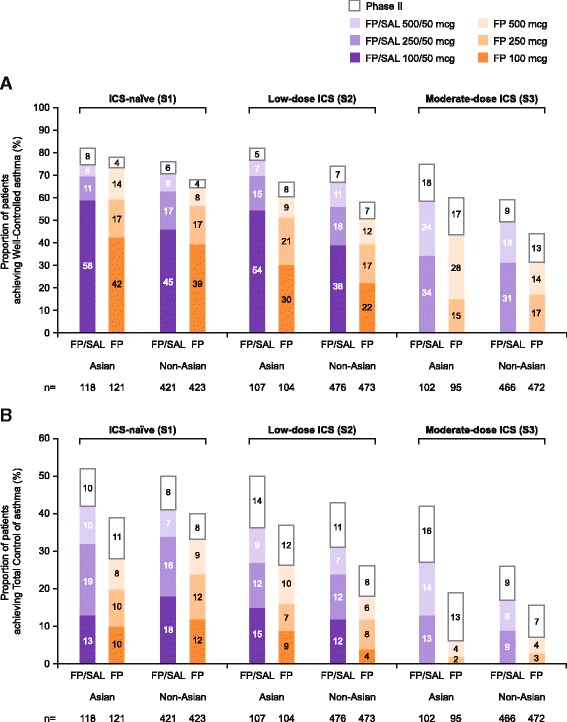
Proportion of patients who achieved Well-Controlled* (**a**) and Total-Control of* (**b**) asthma. *Phase I data are presented by dose and Phase II data are overall proportions only. *FP* fluticasone propionate, *ICS* inhaled corticosteroid, *S1* patients who were ICS naïve at study entry, *S2* patients who received low-dose ICS treatment prior to study entry, *S3* patients who received medium-dose ICS treatment prior to study entry, *SAL* salmeterol

The study also evaluated control according to the ICS dose received. The proportion of Asian patients achieving Well-Controlled asthma and Total Control of asthma was higher with FP/SAL than with FP alone, when FP/SAL was administered at an ICS dose that was the same or lower than the ICS dose with FP alone (Fig. *2*).

More patients (in stratum 1 and stratum 2) achieved Well-Controlled asthma using FP/SAL than FP alone in the Asian population receiving the lowest dose of ICS (FP 100 μg) in Phase I (Fig. *2*a). Findings in the non-Asian population were similar (Fig. *2*a). In the Asian population, the odds of achieving Well-Controlled asthma at the same or lower dose of ICS for FP/SAL versus FP alone in stratum 1 increased by 47% (OR: 1.47; 95% CI: 0.91, 2.38; *p* = 0.119) and more than doubled in stratum 2 (OR: 2.78; 95% CI: 1.64, 4.71; *p* < 0.001) and stratum 3 (OR: 2.38; 95% CI: 1.36, 4.14; *p* = 0.002). Findings of analyses by dose at which control was achieved in the non-Asian population were similar; the use of FP/SAL increased the odds of achieving Well-Controlled asthma compared with FP alone in stratum 1 (OR: 1.37; 95% CI: 1.07, 1.76; *p* = 0.014), stratum 2 (OR: 2.04; 95% CI: 1.61, 2.59; *p* < 0.001), and stratum 3 (OR: 2.20; 95% CI: 1.69, 2.85; *p* < 0.001).

Again, in the Asian population, when analysed by ICS dose at which control was achieved, Total Control of asthma was achieved by a greater proportion of patients using FP/SAL than FP alone (Fig. *2*b). The use of FP/SAL increased the odds of achieving Total Control compared with FP alone (by ICS dose) in stratum 1 (OR: 1.66; 95% CI: 0.97, 2.84; *p* = 0.062), stratum 2 (OR: 1.90; 95% CI: 1.05, 3.46; *p* = 0.035), and stratum 3 (OR: 7.39; 95% CI: 2.74, 19.92; *p* < 0.001). Findings in the non-Asian population were similar; the use of FP/SAL increased the odds of achieving Total Control compared with FP alone in stratum 1 (OR: 1.61; 95% CI: 1.22, 2.12; *p* < 0.001), stratum 2 (OR: 2.06; 95% CI: 1.53, 2.78; *p* < 0.001), and stratum 3 (OR: 2.46; 95% CI: 1.62, 3.72; *p* < 0.001).

#### Control in Phase II

During Phase II of the study, in which patients received a constant dose of medication, additional patients achieved Well-Controlled asthma and Total Control of asthma. The overall proportion of Asian patients achieving Well-Controlled asthma at the end of Phase II was higher with FP/SAL compared with FP alone: 82% (97/118) and 78% (94/121) in stratum 1, 80% (86/107) and 67% (70/104) in stratum 2, and 75% (77/102) and 60% (57/95) in stratum 3 (Fig. 2a). Corresponding values for Total Control of asthma with FP/SAL and FP, respectively, were 52% (61/118) and 39% (47/121) in stratum 1, 50% (54/107) and 37% (38/104) in stratum 2, and 42% (43/102) and 19% (18/95) in stratum 3 (Fig. 2b). A higher proportion of non-Asian patients also achieved control at the end of Phase II with FP/SAL versus FP alone (Fig. 2a and b).

### Analysis of time to control

**Fig. 3:**
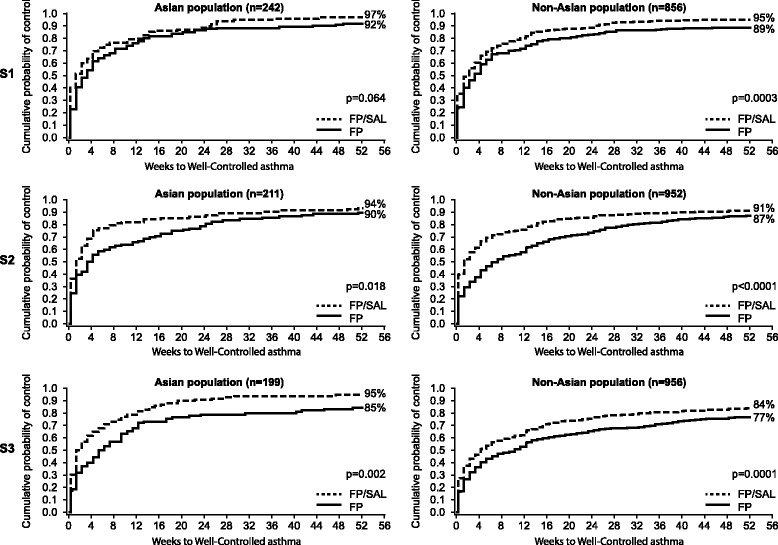
Time to achieve Well-Controlled asthma in Asian and non-Asian patients (S1, S2 and S3), *FP* fluticasone propionate, *ICS* inhaled corticosteroids, *S1* patients who were ICS naïve at study entry, *S2* patients who received low-dose ICS treatment prior to study entry, *S3* patients who received medium-dose ICS treatment prior to study entry, *SAL* salmeterol

**Fig. 4:**
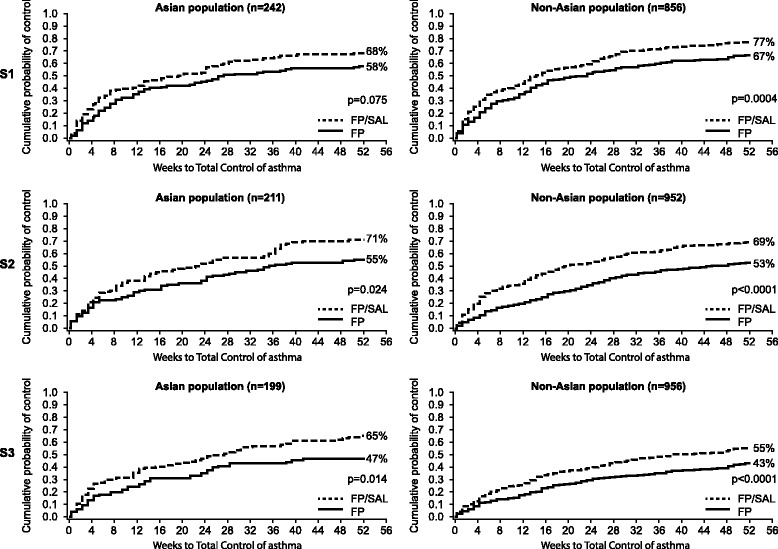
Time to achieve Total-Control of asthma in Asian and non-Asian patients (S1, S2 and S3). *FP* fluticasone propionate, *ICS* inhaled corticosteroids, *S1* patients who were ICS naïve at study entry, *S2* patients who received low-dose ICS treatment prior to study entry, *S3* patients who received medium-dose ICS treatment prior to study entry, *SAL* salmeterol

Analysis of time to control, defined as the time to achieve the first week of Well-Controlled asthma or the first week of Total Control, showed that patients in all groups achieved control more rapidly with FP/SAL than with FP alone, and the cumulative probability of achieving control by Week 52 was greater with FP/SAL than with FP alone (Fig. 3 and Fig. 4).

The cumulative probability of achieving the first week of Well-Controlled asthma by Week 52 with FP/SAL and FP alone, respectively, was 97% (n/N, where N = subgroup total, not the censored value used within the Kaplan–Meier analysis: 115/120) versus 92% (110/122) (*p* = 0.064) for stratum 1, 94% (98/107) versus 90% (92/104) (*p* = 0.018) for stratum 2, and 95% (96/102) versus 85% (79/97) (*p* = 0.002) for stratum 3 in the Asian population (Fig. 3). In the non-Asian population, corresponding values were 95% (392/428) versus 89% (361/428) (*p* < 0.001) for stratum 1, 91% (422/478) versus 87% (393/474) (*p* < 0.001) for stratum 2, and 84% (386/474) versus 77% (349/482) (*p* < 0.001) for stratum 3 (Fig. 3). The time point by which the cumulative probability reached 0.5 in the Asian population for Well-Controlled asthma was 1.3 weeks with FP/SAL versus 3.3 weeks with FP alone in stratum 1, 1.3 weeks versus 3.3 weeks in stratum 2, and 1.8 weeks versus 5.3 weeks in stratum 3. In the non-Asian population, corresponding values were 2.3 weeks versus 3.3 weeks in stratum 1, 1.3 weeks versus 7.3 weeks in stratum 2, and 4.3 weeks versus 10.3 weeks in stratum 3.

For Total Control of asthma, the cumulative probability of patients in the Asian stratum 1 population achieving their first week of control by Week 52 was numerically greater for the FP/SAL group compared with the FP alone group (68% [80/120] vs 58% [69/122], respectively, *p* = 0.075). It was higher in the FP/SAL group than the FP alone group for Asian patients in stratum 2 (71% [73/107] vs 55% [56/104], respectively, *p* = 0.024) and stratum 3 (65% [65/102] vs 47% [43/97], respectively, *p* = 0.014), and in all three strata in non-Asian patients (stratum 1: 77% [311/428] vs 67% [261/428], *p* < 0.001; stratum 2: 69% [311/478] vs 53% [232/474], *p* < 0.001; stratum 3: 55% [247/474] vs 43% [187/482]; *p* < 0.001) (Fig. 4). The time point by which the cumulative probability reached 0.5 in the Asian population for Total Control of asthma was 19.3 weeks with FP/SAL versus 26.3 weeks with FP alone in stratum 1, and 23.3 weeks versus 35.3 weeks in stratum 2. In stratum 3, the first time point of control was achieved by 27.3 weeks with FP/SAL but was not reached with FP. In the non-Asian population, corresponding values were 14.3 weeks versus 23.3 weeks in stratum 1, 20.3 weeks versus 45.3 weeks in stratum 2, and 38.3 weeks versus not reached in stratum 3.

### Stability of control

**Fig. 5:**
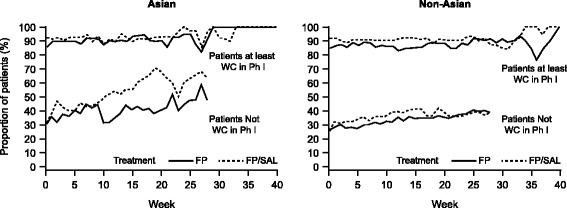
Proportion of patients who achieved at least one week of Well-Controlled asthma during Phase II. Data pooled across all strata within a treatment. This analysis included Phase II treatment only (up to 40 weeks) and control was assessed at each individual week after Phase I. Phase I control status was based on an 8-week assessment period. *FP* fluticasone propionate, *Ph I* Phase I, *SAL* salmeterol, *WC* well controlled

In the Asian population, the majority of patients who achieved Well-Controlled asthma or Total Control of asthma during Phase I (based on an 8-week assessment period) maintained control each week during Phase II (Fig. 5).

In stratum 1, of patients receiving FP/SAL who achieved Well-Controlled asthma during Phase 1 (8-week assessment period), 83% (72/87) had maintained control at the end of Phase II (8-week assessment period) compared with 80% (71/89) receiving FP alone. Corresponding values were 78% (63/81) and 82% (51/62) in stratum 2, and 86% (51/59) and 73% (30/41) in stratum 3 for FP/SAL and FP alone, respectively. For Total Control of asthma, the proportion of patients in the Asian population maintaining control during Phase II with FP/SAL and FP, respectively, were 73% (36/49) and 74% (25/34) in stratum 1, 64% (25/39) and 73% (19/26) in stratum 2, and 78% (21/27) and 83% (5/6) in stratum 3. In the non-Asian population, corresponding values for Well-Controlled asthma were 78% (232/296) and 76% (203/267) in stratum 1, 84% (269/319) and 73% (175/240) in stratum 2, and 75% (172/229) and 78% (115/147) in stratum 3. For Total Control of asthma in the non-Asian population, the proportion maintaining control with FP/SAL and FP, respectively, were 68% (119/176) and 74% (100/135) in stratum 1, 71% (107/150) and 59% (52/88) in stratum 2, and 67% (53/79) and 73% (27/37) in stratum 3. In the Asian population, for patients who did not achieve Well-Controlled asthma or Total Control of asthma in Phase I, a greater proportion of patients using FP/SAL compared with FP gained control in Phase II.

### Effects of treatments on exacerbations

**Fig. 6:**
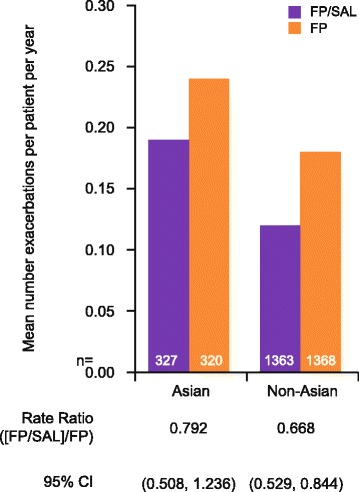
Incidence of exacerbations over 52 weeks. *CI* confidence interval, *FP* fluticasone propionate, *FP/SAL* fluticasone propionate/salmeterol, *ICS* inhaled corticosteroids

As the number of exacerbations reported in the study was low, data were combined across all strata for this analysis. In the Asian population, the incidence of exacerbations over 52 weeks was 0.19 for FP/SAL (*n* = 327) and 0.24 for FP (*n* = 320); rate ratio 0.792 (95% CI: 0.508, 1.236) (Fig. 6). In the non-Asian population, the incidence of exacerbations was 0.12 for FP/SAL (*n* = 1363) and 0.18 for FP (*n* = 1,368); rate ratio 0.668 (95% CI: 0.529, 0.844) (Fig. 6).

### Lung function (FEV_1_)

At Week 52, the change in FEV_1_ was greater with FP/SAL than with FP alone in both the Asian and non-Asian populations: the difference between treatments (FP/SAL minus FP) in adjusted mean change from baseline was 0.22 (95% CI: 0.11, 0.33) in stratum 1, 0.07 (−0.04, 0.18) in stratum 2, and 0.15 (0.04, 0.26) in stratum 3 for the Asian population, and 0.16 (0.10, 0.22) in stratum 1, 0.15 (0.09, 0.20) in stratum 2, and 0.15 (0.09, 0.20) in stratum 3 for the non-Asian population (Additional file 4: Figure S1).

### Asthma QoL

Analysis of AQLQ scores by stratum was limited by few patients completing the questionnaire (in the Asian population, 121 patients treated with FP/SAL and 123 patients with FP alone; in the non-Asian population, 689 patients and 650 patients, respectively, completed the questionnaire); pooled data were therefore analysed. AQLQ scores improved with both FP/SAL and FP alone from baseline to Week 52, in both the Asian and non-Asian populations (Additional file 5: Figure S2).

### Incidence of AEs

The overall incidence of AEs was similar in both treatment groups in both populations. In the Asian population, 192 out of 329 (58%) patients experienced AEs with FP/SAL and 185 out of 323 (57%) with FP alone (Additional file 6: Table S3). Corresponding values in the non-Asian population were 859 out of 1,380 (62%) with FP/SAL and 847 out of 1,384 (61%) with FP alone. The most common AEs reported in both treatment groups were upper respiratory tract infections (in 29–31% of the Asian population, and 9% of the non-Asian population) and nasopharyngitis (in 7–9% of the Asian population, and 14–15% of the non-Asian population) (Additional file 6: Table S3).

## Discussion

This analysis indicated that guideline-defined control can be achieved and maintained in the majority of Asian patients with uncontrolled asthma across a range of severities. More patients in the strata previously receiving a low or medium dose of ICS (stratum 2 + stratum 3) achieved control with FP/SAL compared with FP alone, and did so more quickly and with a lower dose of ICS. For the stratum of patients who were uncontrolled on SABA-only treatment at study entry (stratum 1), there was no difference between the treatments for the primary endpoint of Well-Controlled asthma during the dose-escalation phase. However, there was an advantage for FP/SAL compared with FP alone for Total Control of asthma. This difference could be because Well-Controlled asthma is easier to achieve than Total Control, so treatment differences between FP and FP/SAL are more clearly visible when examining achievement of Total Control; however, the Asian population, although sizeable in this analysis, was too small to examine this observation further. Despite this, a high proportion of patients receiving SABA-only at entry did achieve control. This was accompanied by a noticeable improvement in lung function (0.22 L), as measured by FEV_1_, over the 52 weeks.

During the maintenance phase, additional patients in the Asian population achieved control for both study measures of control compared with the dose-escalation phase. Furthermore, control during the maintenance phase was relatively stable. This benefit of sustained treatment was larger for Total Control than for Well-Controlled asthma across all strata and may reflect additional time required to improve airway inflammation in patients with asthma. Sustained treatment has previously been shown to improve airway hyper-responsiveness over 3 years [16], and it is established that airway hyper-responsiveness is associated with inflammatory markers, such as eosinophilia, in symptomatic patients [17].

As was observed in the total GOAL study population [3], maintaining treatment also had a clear benefit on health status in both the Asian and non-Asian populations studied in this analysis. Insufficient patients in each stratum of the Asian population completed the AQLQ to allow a by-stratum analysis, therefore, the AQLQ data were analysed across in the entire dataset (244 Asian patients in total). The analysis showed that Asian patients experienced a substantial improvement in health status such that the AQLQ score approached or exceeded the value of 6, suggesting that asthma no longer had a significant impact on QoL [18].

The incidence of exacerbations in the Asian patients was low and in keeping with the total study population as previously reported [3]. There was a significant improvement in FEV_1_ for stratum 1 and stratum 3 (0.22 and 0.15 L, respectively), values very similar to those seen in the non-Asian population. The treatment difference for patients in stratum 2 was only 0.07 L.

The findings of the current analysis are in agreement with other studies exploring the efficacy of ICS/LABA combinations in improving asthma control in Asian patients. Although the multinational COSMOS study (1-year, randomised, parallel-group study investigating budesonide/formoterol vs FP/SAL) was conducted in a population that differed from the GOAL population (it included patients with a history of asthma exacerbations in the year prior to entry), a post hoc analysis of the COSMOS Asian population also demonstrated improved asthma control over a 1-year period with two ICS/LABA combinations [5]. Compared with baseline, both combinations provided clinically relevant improvements in asthma control (assessed using the five-item version of the Asthma Control Questionnaire [ACQ]), QoL (assessed using the AQLQ) and FEV_1_ [5]. It should be noted, however, that the ACQ measured asthma control over a recall period of only 7 days, whereas control in the GOAL study was measured over 8-week periods. In another study, the efficacy of a different ICS/LABA combination (fluticasone furoate/vilanterol) was compared with that of FP alone in Asian patients in a 12-week, randomised trial [19]. This ICS/LABA combination provided significantly greater improvements in peak expiratory flow (PEF) compared with FP alone [19]. In a study in Korean patients treated with FP/SAL, the number of patients with Well-Controlled asthma, assessed by the AQLQ and the Asthma Control Test, increased from baseline and was accompanied by a clinically relevant improvement in QoL [20].

Although this is a post hoc analysis of a subgroup of patients from the original, much larger, population [3], it is reassuring to see that existing and well-established treatments are as effective in Asian patients as in a wider group of patients at achieving and maintaining comprehensive control of asthma, and that this is accompanied by additional outcome benefits.

Safety findings from the present analysis were also similar with the findings from the other studies, with the most common AEs reported with ICS/LABA combinations or ICS alone being upper respiratory tract infection (URTI) and nasopharyngitis [5, 19, 20]. A substantial difference was noted between Asian and non-Asian patients in the incidence of these AEs: ~30% of Asian patients experienced URTI compared with only 9% of non-Asian patients, whilst fewer Asian patients (8%) experienced nasopharyngitis than non-Asian patients (14%). Differences in patterns of antibiotic prescription in Asian and non-Asian countries may explain the difference in the incidence of URTI between the two populations, but could not account for the discrepancy in nasopharyngitis. An alternative explanation may simply be that different terms are used in the different countries included within the populations to describe the same symptoms.

This analysis suggests that, in Asian patients, baseline lung function may not be an accurate predictor of future asthma control. While asthma severity is related to FEV_1_ [21] and ethnic differences in lung function (including FEV_1_) have also been reported [10, 11], it is unclear how these different factors are linked. Lung function also varies with gender and age [11, 22], and so further research into the factors determining severity and differences in baseline physiology in Asians compared with non-Asians is required.

Strengths of this study included the large number of Asian patients within the analyses and the use of the guideline-defined composite endpoints of asthma control. A number of limitations should be taken into account when considering these findings. This was a post hoc subgroup analysis and was not designed to analyse treatment differences in asthma control within and between the Asian and non-Asian populations. The Asian subgroup population was also much smaller (N = 652) than the non-Asian subgroup population (N = 2,764), and, therefore, within and between subgroup comparisons must be interpreted with caution. This difference in sample size may have impacted some of the results. For example, in Asian patients who were ICS-naïve at baseline, there was no difference between FP/SAL and FP alone with regard to the proportion achieving Well-Controlled asthma, but a benefit of FP/SAL over FP alone was observed in this group for Total Control of asthma. In the corresponding group of non-Asian patients, a benefit for FP/SAL was seen for both endpoints. Patient numbers were too small to enable analyses by stratum for some outcomes (i.e. AQLQ); only pooled data were presented for these parameters. Although this Asian subgroup is one of the largest populations studied with regard to a composite score of asthma control, it was not large enough to conduct a by-stratum analysis of exacerbations, an important outcome for patients with asthma. Finally, the Asian patient population included in this analysis was heterogeneous as it included patients from several countries. This may limit generalisability to more homogeneous, single country populations.

## Conclusions

The results of the GOAL study in Asian patients reflected those seen in the overall population [3]. In addition, the present analysis demonstrated similar trends in Asian compared with non-Asian patients. The post hoc subgroup analyses showed that guideline-defined asthma control, measured by a composite of clinically relevant endpoints, can be achieved and maintained with the combination of FP/SAL in a substantial proportion of Asian patients. Overall, FP/SAL was more effective at a lower dose of ICS than FP alone in achieving control and achieved control more quickly than FP alone.

## Additional files

Additional file 1: Supplementary materials. Study oversight and study population. (DOC 36 kb)

Additional file 2: Table S1. Definitions of Total Control of asthma and Well-Controlled asthma, modified from the GINA/NIH Guidelines. (DOCX 25 kb)

Additional file 3: Table S2. Patient race (ITT population). (DOCX 25 kb)

Additional file 4: Figure S1. Lung function (FEV_1_) at Phase II endpoint. (DOCX 79 kb)

Additional file 5: Figure S2. Asthma quality of life questionnaire score (pooled data) in Asian and non-Asian patients, at baseline and after 52 weeks of treatment. (DOCX 112 kb)

Additional file 6: Table S3. Summary of the most common adverse events (≥5%) in the Asian and non-Asian populations. (DOCX 24 kb)

## Abbreviations

ACQ: Asthma Control Questionnaire
AEs: Adverse events
AIRIAP: Asthma Insights and Reality in Asia-Pacific survey
am: Morning
AQLQ: Asthma Quality of Life Questionnaire
BMI: Body mass index
CI: Confidence interval
ECCS: European Community for Coal and Steel
FEV_1_: Forced expiratory volume in 1 s
FP: Fluticasone propionate
GINA: Global Initiative for Asthma
GOAL: Gaining Optimal Asthma controL
ICS: Inhaled corticosteroid
ITT: Intent-to-treat
LABA: Long-acting β_2_-agonist
max.: Maximum
min.: Minimum
NIH: National Institutes of Health
OR: Odds ratio
PEF: Peak expiratory flow
QoL: Quality of life
REALISE: REcognise Asthma and LInk to Symptoms and Experience study
S: Stratum
SABA: Short-acting β2-agonist
SAL: Salmeterol
SD: Standard deviation
URTI: Upper respiratory tract infection
WC: Well controlled
