# Supplementary material for: Asthma control using fluticasone propionate/salmeterol in Asian and non-Asian populations: a post hoc analysis of the GOAL study
Source: BMC Pulm Med. 2017 Apr 28;17:75. doi: 10.1186/s12890-017-0410-x (PMC5410062; doi:10.1186/s12890-017-0410-x)
Supplement: Supplementary file 1 — Supplementary materials. Study oversight and study population. (DOC 36 kb) [file 12890_2017_410_MOESM1_ESM.doc]

# Supplementary Materials

# Methods

# Study oversight

The original study was approved by local research ethics committees, and all patients or their guardians gave written informed consent.

Study population

Patients were 12–80 years of age, with: a history of asthma for ≥6 months; a ≥15% improvement in forced expiratory volume in 1 second (FEV_1_) on inhalation of a short-acting β_2_-agonist; a smoking history of <10 pack-years; no use of long-acting β_2_-agonists in the 2 weeks prior to the study; and a ≥2-week period of not Well-Controlled asthma in the 4-week run-in period [1].

# References

[1] E.D. Bateman, H.A. Boushey, J. Bousquet, W.W. Busse, T.J. Clark, R.A. Pauwels, S.E. Pedersen, G.I. Group, Can guideline-defined asthma control be achieved? The Gaining Optimal Asthma ControL study, Am. J. Respir. Crit. Care Med. 170 (2004) 836-44.
