# Supplementary material for: Asthma control using fluticasone propionate/salmeterol in Asian and non-Asian populations: a post hoc analysis of the GOAL study
Source: BMC Pulm Med. 2017 Apr 28;17:75. doi: 10.1186/s12890-017-0410-x (PMC5410062; doi:10.1186/s12890-017-0410-x)
Supplement: Supplementary file 2 — Definitions of Total Control of asthma and Well-Controlled asthma, modified from the GINA/NIH Guidelines. (DOCX 25 kb) [file 12890_2017_410_MOESM2_ESM.docx]

**Table S1.** Definitions of Total Control of asthma and Well-Controlled asthma, modified from the GINA/NIH Guidelines [1,2]

|  | **Total Control**  **Each week all of:** | **Well-Controlled**  **Each week, ≥2 of:** |
| --- | --- | --- |
| Daytime symptoms | None | ≤2 days with symptom score >1^a^ |
| Rescue β_2_-agonist use | None | Use on ≤2 days and ≤4 occasions/week |
| Morning PEF | ≥80% predicted every day | ≥ 80% predicted every day |
|  |  | All of: |
| Night-time awakening | None | None |
| Exacerbations^b^ | None | None |
| Emergency visits | None | None |
| Treatment-related AEs | None enforcing change in asthma therapy | None enforcing change in asthma therapy |

Total Control of asthma and Well-Controlled asthma were defined by achievement of all of the specified criteria for that week. Total Control of asthma was achieved if the patient during the
8 consecutive assessment weeks recorded 7 weeks with Total Control and had no exacerbations, emergency room criteria, or medication-related adverse events criteria. Well-Controlled asthma was similarly assessed over the 8 weeks. These assessments were for an 8-week period during the double-blind treatment period. Baseline control and control during the open-label phase were assessed over a 4-week period.

^a^Symptom score: 1 was defined as “symptoms for one short period during the day.” Overall scale: 0 (none)–5 (severe).

^b^Exacerbations were defined as deterioration in asthma requiring treatment with an oral corticosteroid or an emergency department visit or hospitalisation.

AE, adverse event; GINA/NIH, Global Initiative for Asthma/National Institutes of Health; PEF, peak expiratory flow.

References

[1] E.D. Bateman, H.A. Boushey, J. Bousquet, W.W. Busse, T.J. Clark, R.A. Pauwels, S.E. Pedersen, G.I. Group, Can guideline-defined asthma control be achieved? The Gaining Optimal Asthma ControL study, Am. J. Respir. Crit. Care Med. 170 (2004) 836-44.

[2] GINA/NIH,Global Strategy for Asthma Management and Prevention, 2002. Available from: <http://www.ginasthma.org/local/uploads/files/GINAwr02.pdf> [accessed February 22, 2016].
