# Supplementary material for: Asthma control using fluticasone propionate/salmeterol in Asian and non-Asian populations: a post hoc analysis of the GOAL study
Source: BMC Pulm Med. 2017 Apr 28;17:75. doi: 10.1186/s12890-017-0410-x (PMC5410062; doi:10.1186/s12890-017-0410-x)
Supplement: Supplementary file 3 — Patient race (ITT population). (DOCX 25 kb) [file 12890_2017_410_MOESM3_ESM.docx]

**Table S2.** Patient race (ITT population)

|  | **S1** | | | **S2** | | | **S3** | |
| --- | --- | --- | --- | --- | --- | --- | --- | --- |
|  | **FP/SAL** | **FP** | **FP/SAL** | | **FP** | **FP/SAL** | | **FP** |
| **Asian population, n (%)** | n=120 | n=122 | n=107 | | n=104 | n=102 | | n=97 |
| Chinese | 60 (50) | 62 (51) | 66 (62) | | 65 (63) | 43 (42) | | 42 (43) |
| Thai | 29 (24) | 28 (23) | 13 (12) | | 13 (13) | 19 (19) | | 17 (18) |
| Malay | 1 (<1) | 1 (<1) | 4 (4) | | 2 (2) | 1 (<1) | | 1 (1) |
| Indian subcontinent | 0 | 1 (<1) | 1 (<1) | | 0 | 2 (2) | | 1 (1) |
| Japanese | 1 (<1) | 0 | 0 | | 0 | 0 | | 0 |
| Korean | 10 (8) | 11 (9) | 12 (11) | | 14 (13) | 28 (27) | | 29 (30) |
| Filipino | 19 (16) | 19 (16) | 11 (10) | | 10 (10) | 4 (4) | | 3 (3) |
| Other Asian | 0 | 0 | 0 | | 0 | 5 (5) | | 4 (4) |
| **Non-Asian population, n (%)** | n=428 | n=428 | n=478 | | n=474 | n=474 | | n=482 |
| White | 378 (88) | 372 (87) | 421 (88) | | 422 (89) | 420 (89) | | 424 (88) |
| Black | 14 (3) | 16 (4) | 8 (2) | | 2 (<1) | 12 (3) | | 10 (2) |
| Asian | 7 (2) | 5 (1) | 6 (1) | | 9 (2) | 6 (1) | | 10 (2) |
| American Hispanic | 23 (5) | 32 (7) | 38 (8) | | 36 (8) | 31 (7) | | 36 (7) |
| Other | 6 (1) | 3 (<1) | 5 (1) | | 5 (1) | 5 (1) | | 2 (<1) |

The Asian population was defined as patients of Chinese, Japanese, Korean, Thai, Malay or Filipino origin living in South East Asia (China, Taiwan, Singapore, Hong Kong, Thailand, Philippines, South Korea, Malaysia, Japan) during the study. The non-Asian population included all patients in the ITT population who were not in the Asian subgroup.

FP, fluticasone propionate; ITT, intent-to-treat; S1, patients who were ICS naïve at study entry; S2, patients who received low-dose ICS treatment prior to study entry; S3, patients who received medium-dose ICS treatment prior to study entry; SAL, salmeterol.
