# Supplementary material for: Asthma control using fluticasone propionate/salmeterol in Asian and non-Asian populations: a post hoc analysis of the GOAL study
Source: BMC Pulm Med. 2017 Apr 28;17:75. doi: 10.1186/s12890-017-0410-x (PMC5410062; doi:10.1186/s12890-017-0410-x)
Supplement: Supplementary file 4 — Lung function (FEV1) at Phase II endpoint. (DOCX 79 kb) [file 12890_2017_410_MOESM4_ESM.docx]

**Figure S1.** Lung function (FEV_1_) at Phase II endpoint


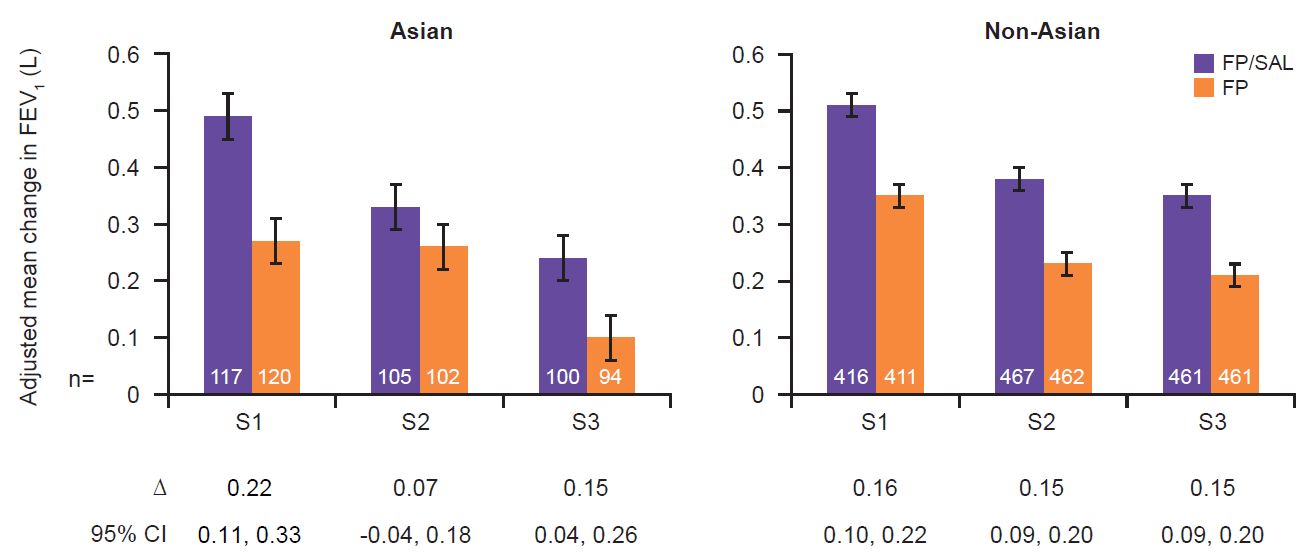


Error bars represent the standard error.

CI, confidence interval; Δ, change between FP/SAL and FP; FEV_1_, forced expiratory volume in 1 second; FP, fluticasone propionate; ICS, inhaled corticosteroids; S1, patients who were ICS naïve at study entry; S2, patients who received low-dose ICS treatment prior to study entry; S3, patients who received medium-dose ICS treatment prior to study entry; SAL, salmeterol.
