# Supplementary material for: Asthma control using fluticasone propionate/salmeterol in Asian and non-Asian populations: a post hoc analysis of the GOAL study
Source: BMC Pulm Med. 2017 Apr 28;17:75. doi: 10.1186/s12890-017-0410-x (PMC5410062; doi:10.1186/s12890-017-0410-x)
Supplement: Supplementary file 5 — Asthma quality of life questionnaire score (pooled data) in Asian and non-Asian patients, at baseline and after 52 weeks of treatment. (DOCX 112 kb) [file 12890_2017_410_MOESM5_ESM.docx]

**Figure S2.** Asthma quality of life questionnaire score (pooled data) in Asian and non-Asian patients, at baseline and after 52 weeks of treatment


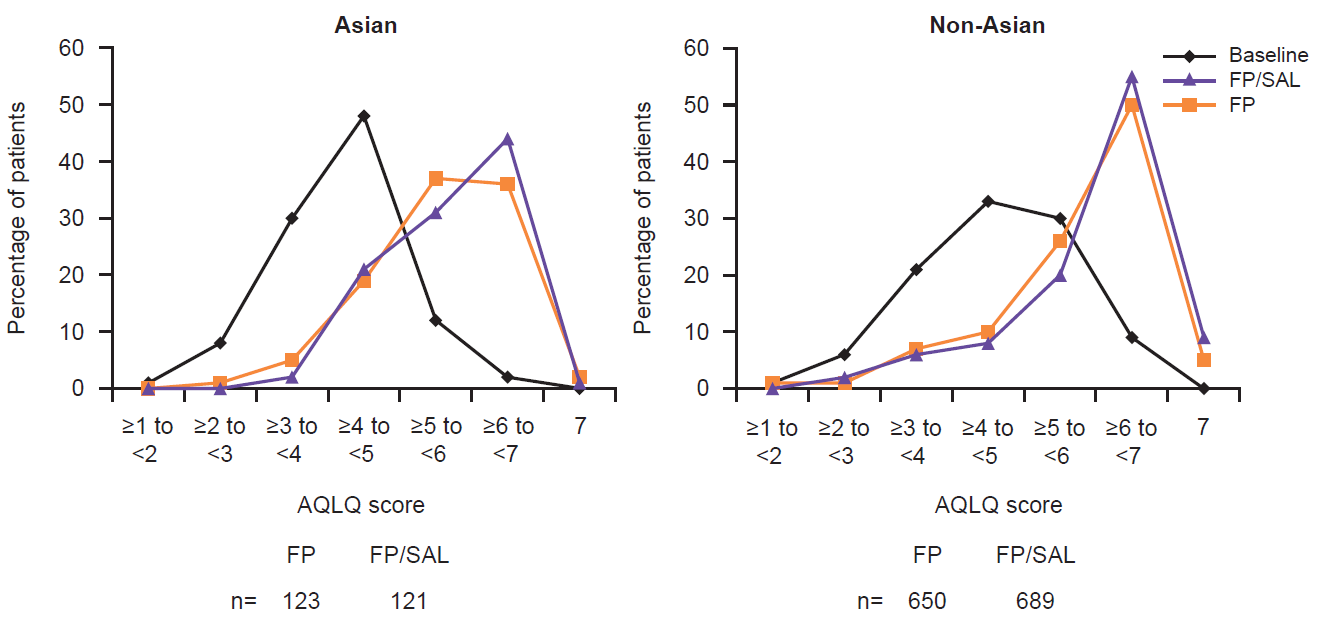


AQLQ, Asthma Quality of Life Questionnaire; FP, fluticasone propionate; FP/SAL, fluticasone propionate/salmeterol; n, those who completed the AQLQ questionnaire.
