# Supplementary material for: Asthma control using fluticasone propionate/salmeterol in Asian and non-Asian populations: a post hoc analysis of the GOAL study
Source: BMC Pulm Med. 2017 Apr 28;17:75. doi: 10.1186/s12890-017-0410-x (PMC5410062; doi:10.1186/s12890-017-0410-x)
Supplement: Supplementary file 6 — Summary of the most common adverse events (≥5%) in the Asian and non-Asian populations. (DOCX 24 kb) [file 12890_2017_410_MOESM6_ESM.docx]

**Table S3.** Summary of the most common adverse events (≥5%) in the Asian and non-Asian populations

|  |  | **Asian population** | | **Non-Asian population** | |
| --- | --- | --- | --- | --- | --- |
|  |  | **FP/SAL (N=329)** | **FP (N=323)** | **FP/SAL (N=1,380)** | **FP (N=1,384)** |
| Any event, n (%) | | 192 (58) | 185 (57) | 859 (62) | 847 (61) |
| Upper respiratory tract infection | | 97 (29) | 99 (31) | 125 (9) | 118 (9) |
| Nasopharyngitis | | 24 (7) | 29 (9) | 197 (14) | 203 (15) |
| Cough | | 18 (5) | 18 (6) | - | - |
| Headache | | 10 (3) | 25 (8) | 82 (6) | 94 (7) |
| Hoarseness | | 15 (3) | 10 (3) | - | - |
| Sinusitis | | - | - | 83 (6) | 71 (5) |
| Influenza | | - | - | 75 (5) | 64 (5) |

FP, fluticasone propionate; SAL, salmeterol.
